# Likelihood estimation for a longitudinal negative binomial regression model with missing outcomes

**DOI:** 10.1111/j.1467-9876.2008.00651.x

**Published:** 2009-07

**Authors:** Simon J Bond, Vernon T Farewell

**Affiliations:** Mundipharma Research LimitedCambridge, UK; Medical Research Council Biostatistics UnitCambridge, UK

**Keywords:** Informative observation, Likelihood, Missing data, Psoriatic arthritis, Regression, Simulation

## Abstract

Joint damage in psoriatic arthritis can be measured by clinical and radiological methods, the former being done more frequently during longitudinal follow-up of patients. Motivated by the need to compare findings based on the different methods with different observation patterns, we consider longitudinal data where the outcome variable is a cumulative total of counts that can be unobserved when other, informative, explanatory variables are recorded. We demonstrate how to calculate the likelihood for such data when it is assumed that the increment in the cumulative total follows a discrete distribution with a location parameter that depends on a linear function of explanatory variables. An approach to the incorporation of informative observation is suggested. We present analyses based on an observational database from a psoriatic arthritis clinic. Although the use of the new statistical methodology has relatively little effect in this example, simulation studies indicate that the method can provide substantial improvements in bias and coverage in some situations where there is an important time varying explanatory variable.

## 1. Introduction and motivation

This work was motivated by on-going analyses of a longitudinal database of information on patients with the chronic condition psoriatic arthritis (Hanley *et al.*, 1988). This database, which was initiated in 1978, derives from the psoriatic arthritis clinic at the Toronto Hospital and records information from visits to the clinic which occur approximately once every 6 months. The principal measure of a patient's disease progression is the number of damaged joints. Damage to a joint is a permanent condition, typically reflecting immobility, and can be contrasted with disease activity, which is reflected by pain and inflammation of joints, which can often be alleviated by treatment.

There are *two* principal ways to measure joint damage: through a clinical examination and clinician assessment of damage, and through assessment of X-rays of joints, this typically being restricted to hands and feet. The clinical examination can be performed on every clinic visit but is often considered more subjective than the radiological examination, which is generally undertaken at intervals of approximately 2 years. More frequent routine X-rays would not be clinically acceptable owing to safety and resource implications.

Past studies of disease progression (e.g. Gladman and Farewell (1999), Gladman *et al.* (1994) and Bond *et al.* (2007)) have used the number of damaged joints developing between visits to the clinic as a primary outcome measure where the joint counts are those determined by clinical examination. The increment in joint counts has been related to explanatory variables, with a particular interest in the relationship between time varying explanatoryvariables reflecting disease activity and the subsequent occurrence of damage. With complete data, where we observe the damaged joint count and all explanatory variables of interest at each clinic visit, it is straightforward to compute the increment in the number of damaged joints and to fit a relevant model, such as a Poisson or negative binomial regression model.

After 30 years of data collection, the next phase of data analysis will focus on predictors of clinical damage at the individual joint level rather than at the patient level. Before initiating this work, it was felt important to provide as much evidence as possible that findings based on clinical damage are informative about disease progression and, in particular, would be consistent with those that might have been derived through use of radiological measures of damage if these were as frequently available. There is now enough evidence to consider this for patient level measurements and the work that is reported here was motivated by the desire to examine patient level predictors of radiological damage to confirm earlier findings that were based on clinical damage. The situation that arises in such analyses is that we have information on time varying explanatory variables being collected at each visit to the clinic but the primary radiological outcome measure is updated less frequently.

One approach is to consider only the data that are collected on the clinic visits when a radiograph is taken. This enables a regression model to be fitted using standard software for the implementation of generalized linear models (McCullagh and Nelder, 1989) and was done by Bond *et al.* (2007). However, it may be desired, and felt to be more sensible, to use the updated information on explanatory variables that is available from the intermediate clinic visits when a radiograph has not been taken. The primary aim of this paper is to present a means to implement a likelihood method for performing such estimation and to consider how much difference there is between the first ‘naive’ but straightforward method and the second more comprehensive approach to the analysis of the radiological outcome data. If consistent results are found between these two methods for the modelling of radiological data, then, given the comparable results that were found by Bond *et al.* (2007), reassurance is provided concerning the use of clinical measures of damage in the analysis of data at the individual joint level. Although, in this application, consistency of results between the two analyses of radiological data is a particular advantage, in other settings there might be the expectation that the more complicated analysis will differ from and be more appropriate than the naive method. Of course, if this were true for the psoriatic arthritis data, the more important question clinically would be whether the appropriate analysis of the radiological damage is consistent with the analysis of the clinical damage.

Section 2 defines some general notation and presents the technical details for dealing with missing outcome data. We perform some simulations in Section 3 to determine how much the analysis suggested can differ from a naive analysis in situations that are characterized by the influence and variability of an explanatory variable as well as the pattern of missingness. Section 4 outlines very briefly how informative observation might be incorporated into the likelihood methodology. Section 5 applies the method to the data that motivated the work. In addition, models for clinical damage are examined so that by deleting observations we can check how the method compares with the use of complete data with no missing outcomes. The paper concludes with a discussion in Section 6.

## 2. Methodology

Assume that each patient *i* has *m*_*i*_ clinic visits (*m*_*i*_≥2), and there are *n* patients. At each visit to the clinic there is, potentially unobserved, a variable *D*_*i*,*j*_ the number of damaged joints, and a vector of explanatory variables **X**_*i*,*j*_, where 1≤*i*≤*n* and 1≤*j*≤*m*_*i*_. Define the response variable *Y*_*i*,*j*_=*D*_*i*,*j*+1_−*D*_*i*,*j*_,1≤*j*<*m*_*i*_, so the current set of explanatory variables is used to predict the *next* increment in the damaged joint count. Note that the number of increments *Y*_*i*,*j*_ observed is one fewer than the number of visits at which the joint counts *D*_*i*,*j*_ are observed and that there is no restriction that the damaged joint count at the first clinic visit, *D*_*i*,0_, be 0. We could allow the explanatory variables **X**_*i*,*j*_ to include functions of the damaged joint counts observed up to and including visit *j*. For simplicity, however, we exclude this possibility initially and extend the methodology to permit it subsequently.

We assume that 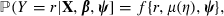 where *f*{·} defines a probability distribution function, on the integers, and has a finite number of parameters. The location parameter is assumed to be a known function *μ*(·) of a linear combination of the explanatory variables plus any offset terms, *η*=**X*β***+**O**, and any further nuisance parameters are given by ***ψ***. In our motivating example, *f* is the negative binomial distribution, *μ* is the exponential function and *ψ* is a single nuisance parameter representing overdispersion. If all the *D*_*i*,*j*_, and consequently all the *Y*_*i*,*j*_, were observed then we can define the likelihood function to be 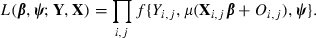
(1)

In contrast, consider the case where we observe (dropping the *i*-suffix) **X**_1_,**X**_2_ and **X**_3_, the explanatory variables on visits 1, 2 and 3, but we observe only *D*_1_ and *D*_3_ with the second visit missing. In this case we can only infer that 0≤*Y*_1_≤*D*_3_−*D*_1_, 0≤*Y*_2_≤*D*_3_−*D*_1_ and *Y*_1_+*Y*_2_=*D*_3_−*D*_1_. Provided that the observation pattern corresponds to the *Y*s being missing at random (Rubin, 1976), the likelihood function can be written as 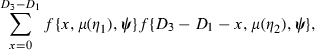 where *η*_*j*_=**X**_*j*_***β***+*O*_*j*_. The fact that the random variable *Y*_2_ has a finite and discrete sample space means that we can calculate the likelihood accurately, rather than having to approximate an integral, which would be the case if the state space was continuous.

Where there is a sequence of multiple missing observations, it is helpful to think of the idea of a multistate model (Cox and Miller, 1965; Billingsley, 1981). Define the states to be the number of damaged joints. If we observe **X**_1_,…,**X**_*k*−1_ and only *D*_1_ and *D*_*k*_ then we know that the unobserved sequence of states must be an increasing sequence starting at *D*_1_ and finishing at *D*_*k*_. Modelling assumptions provide us with the transition probabilities. These are defined to be the matrices **P**^*j*^,1≤*j*≤*k*−1, with elements 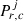 (*r*,*c* meaning ‘row–column’). 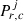 is the probability that the patient goes to state *c* at visit *j*+1 conditional on their being in state *r* at visit *j*, so 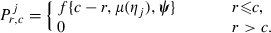
(2)

For the first transition, **P**^1^ is a row vector, since we have observed that *r*=*D*_1_, rather than a range of possible values. For the final transition, **P**^*k*−1^ is a column vector, since we have observed that *c*=*D*_*k*_. For the remaining values of *j* (1<*j*<*k*−1) **P**^*j*^ is a square matrix with *D*_*k*_−*D*_1_+1 rows and columns, since *r* and *c* both can take values from {*D*_1_,*D*_1_+1,…,*D*_*k*_−1,*D*_*k*_}. The likelihood is the matrix product, 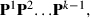 which is a scalar number since the first and last matrices are row and column vectors respectively.

Hitherto, we assumed that **X**_*j*_ did not include any of the values of the response variable observed at current or previous visits. When calculating the transition probability matrix **P**^*j*^ for a set of parameter values, this allows us to calculate *μ*(*η*_*j*_) just once, and then the vector of values of *f*{·} can be calculated for just the first row of **P**^*j*^, and then reused (without any further calculations) in the subsequent rows. This helps to ease the computational cost. However, if we want to include functions of the current, but potentially unobserved, value of *D*_*j*_ at transition *j*, which is denoted as **Z**(*D*_*j*_), then an extension of equation (2) is required. A motivation for this is to take account of correlation. In this case, we modify the location parameter *μ*(*η*_*j*_) to become 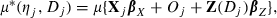 and modify equation (2) to be 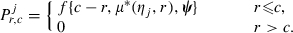
(3)

The computational cost of this is that to calculate **P**^*j*^ now requires a separate evaluation of *f*{·} with different arguments for every non-zero entry of **P**^*j*^.

We have defined the likelihood contribution for every sequence of contiguous missing values that are bounded by observed values. Two or more successive observed values give a simple product of probabilities, similar to equation (1). If the patient's records finish with a sequence of contiguous missing values then these observations are discarded since they give no information. The complete likelihood for the entire data is the product over visits and patients of all such terms. The complete likelihood is then used to derive maximum likelihood estimates or a posterior distribution if combined with prior distributions on the parameters. If the likelihood is maximized by using a Newton-type method (Dennis and Schnabel, 1983) the approximation to the Hessian matrix is used to estimate the covariance matrix for the parameter estimates and subsequently to form confidence intervals that are based on the asymptotic normality of maximum likelihood estimates.

## 3. Simulation

For our simulations, we generated independent outcome variables *Y* from a negative binomial distribution, with dispersion parameter 1, and with the linear predictor 1+*X* *β*. The coefficient *β* took values 1 or 2; the explanatory variable *X* was a binary variable with values 0 or 1. This implicitly assumes equidistant observations. Our first simulation held *X* constant within a patient and hence is comparable with a randomized control trial. This situation acts as a validation for the simulation in that little gain can be expected when explanatory variables are not time varying. We generated data for 10 ‘patients’, five of whom had *X*=1, and each patient had 10 observations. The situation of 10 patients is not of practical interest but is used to illustrate the nature of possible effects and a more realistic scenario is examined subsequently. To study the effect of the amount of missingness, we selected at random, without replacement, a sample of fixed size from the integers {2,3,…,9}. These observation numbers were then deleted from the simulated data, and only the sum of the relevant *Y*s retained (we ensured that the first and last observations were never deleted). The simulated data were then analysed in three ways: using all the original data and standard software to give a benchmark for comparison, using the missing data and standard software where the extra information that is provided by additional observations of explanatory variables was just ignored and using the missing data and the new methods of Section 2.

For each model specification, 500 simulations were undertaken and the maximum likelihood estimates and associated 95% confidence intervals were recorded. From these, the mean-squared error, the bias and coverage of the three parameter estimates (intercept, *β* and dispersion) were estimated. Table 1, part (a), presents selected results of these simulations, tabulated by the number of missing observations and by method.

**Table 1:** Simulation results

*Method*	*Missing*	*Bias*			*Mean-squared error*			*Coverage*		
		*Dispersion*	*Intercept*	*β*	*Dispersion*	*Intercept*	*β*	*Dispersion*	*Intercept*	*β*
*(a) Patient constant explanatory variable*										
Full	2.000	0.061	−0.012	0.001	0.047	0.026	0.051	0.958	0.950	0.960
New	2.000	0.085	−0.016	0.004	0.069	0.030	0.059	0.962	0.960	0.952
Naive	2.000	0.328	−0.014	0.001	0.205	0.031	0.062	0.942	0.966	0.958
Full	3.000	0.058	−0.018	0.010	0.049	0.033	0.054	0.944	0.964	0.954
New	3.000	0.101	−0.020	0.008	0.088	0.038	0.061	0.970	0.946	0.948
Naive	3.000	0.535	−0.021	0.006	0.448	0.043	0.069	0.891	0.944	0.954
Full	4.000	0.048	−0.003	−0.011	0.046	0.033	0.068	0.953	0.943	0.941
New	4.000	0.123	−0.005	−0.008	0.133	0.036	0.076	0.963	0.943	0.923
Naive	4.000	0.825	−0.011	0.001	1.063	0.043	0.088	0.843	0.941	0.921
Full	5.000	0.051	−0.001	−0.012	0.044	0.031	0.056	0.961	0.959	0.955
New	5.000	0.141	−0.006	−0.010	0.152	0.034	0.063	0.969	0.953	0.945
Naive	5.000	1.270	−0.012	−0.003	2.280	0.038	0.073	0.761	0.955	0.947
*(b) Explanatory variable with positive dependence*										
Full	2.000	0.054	−0.011	−0.010	0.049	0.026	0.052	0.970	0.940	0.934
New	2.000	0.069	−0.010	−0.013	0.064	0.029	0.059	0.970	0.940	0.926
Naive	2.000	0.249	0.030	−0.076	0.145	0.030	0.065	0.954	0.940	0.936
Full	3.000	0.037	−0.018	0.003	0.038	0.027	0.051	0.974	0.936	0.952
New	3.000	0.062	−0.017	0.001	0.062	0.030	0.058	0.966	0.942	0.946
Naive	3.000	0.363	0.042	−0.099	0.236	0.032	0.069	0.938	0.930	0.926
Full	4.000	0.058	−0.018	0.002	0.045	0.029	0.048	0.962	0.930	0.948
New	4.000	0.099	−0.015	−0.004	0.080	0.035	0.061	0.972	0.932	0.948
Naive	4.000	0.547	0.075	−0.154	0.442	0.040	0.084	0.888	0.894	0.886
Full	5.000	0.048	−0.026	0.004	0.041	0.029	0.054	0.968	0.926	0.936
New	5.000	0.122	−0.027	0.003	0.112	0.039	0.071	0.974	0.930	0.944
Naive	5.000	0.797	0.093	−0.185	1.033	0.047	0.104	0.852	0.872	0.854

As expected, all three methods have minimal bias and adequate coverage for the location term and the regression coefficient which should be estimated consistently by all methods. The same patterns were seen for both values of *β* and therefore results only for *β*=1 are presented here and subsequently. If data were simulated according to a Poisson distribution, and Poisson regression models were used, the naive method and our likelihood method would coincide since the explanatory variable *x* is constant within patients and the sum of Poisson distributions is Poisson. The negative binomial distribution can be considered as a Poisson distribution with a multiplicative random effect whose variance is a function of the dispersion parameter. Hence it is not surprising that the naive method performs reasonably well for the coefficients (when associated with patient constant explanatory variables) but not the dispersion. Thus, although the naive method is biased for estimation of the dispersion parameter of the generating negative binomial, it does provide consistent estimation of the appropriate dispersion parameter for the naive analysis. The difference between these dispersion parameters increases as the number of missing observations increases. With observations that are not equidistant, the naive method would also involve some lack of fit since the sum of negative binomials would not be a negative binomial in this situation but the effect might be minimal in practice.

Tables 1, part (b), and 2, part (a), present the results of additional simulation exercises. These were similar to the first two; however, the difference was that the explanatory variable *x* varied within patients. This is the situation in which the naive method has the potential to give misleading results. The set of explanatory variables was simulated according to the distributions 
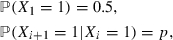

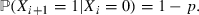


The parameter *p* was set to 0.8 to generate a set of *X*-values that had a positive dependence, and with *p*=0.2 for a negative dependence. These two patterns of explanatory variables were then held constant over 500 simulations.

The results demonstrate that the naive method performs poorly for all parameters, with increasing bias with increased missingness. Our likelihood method is unbiased and gives good coverage. The effect of the missingness is to increase the mean-squared error.

Given that the simulations with a negative dependence produced the largest bias in the naive method, we choose to consider a large sample simulation using the same ‘true’ model. We repeated the simulation but with 100 patients rather than 10, each patient with 10 observations and with the same patterns of missingness as used previously. The results are shown in Table 2, part (b).

**Table 2:** Simulation results: explanatory variable with negative dependence

*Method*	*Missing*	*Bias*			*Mean-squared error*			*Coverage*		
		*Dispersion*	*Intercept*	*β*	*Dispersion*	*Intercept*	*β*	*Dispersion*	*Intercept*	*β*
*(a) 10 patients*										
Full	2.000	0.052	−0.004	−0.010	0.041	0.026	0.044	0.954	0.952	0.962
New	2.000	0.069	−0.014	0.001	0.060	0.035	0.064	0.950	0.958	0.960
Naive	2.000	0.186	0.125	−0.202	0.094	0.047	0.096	0.970	0.870	0.860
Full	3.000	0.054	−0.020	0.006	0.046	0.031	0.052	0.958	0.950	0.942
New	3.000	0.093	−0.033	0.020	0.074	0.050	0.088	0.970	0.934	0.946
Naive	3.000	0.309	0.177	−0.283	0.186	0.066	0.141	0.944	0.790	0.750
Full	4.000	0.055	−0.014	−0.002	0.043	0.029	0.056	0.952	0.946	0.918
New	4.000	0.092	−0.032	0.014	0.078	0.063	0.118	0.968	0.938	0.936
Naive	4.000	0.431	0.231	−0.364	0.301	0.087	0.200	0.942	0.754	0.678
Full	5.000	0.041	−0.019	0.008	0.038	0.028	0.054	0.964	0.954	0.934
New	5.000	0.099	−0.037	0.021	0.104	0.079	0.146	0.964	0.950	0.946
Naive	5.000	0.617	0.289	−0.449	0.593	0.117	0.268	0.858	0.687	0.585
*(b) 100 patients*										
Full	2.000	0.007	−0.002	0.004	0.003	0.002	0.005	0.962	0.966	0.960
New	2.000	0.009	−0.001	0.004	0.004	0.003	0.006	0.960	0.974	0.960
Naive	2.000	0.119	0.152	−0.207	0.019	0.026	0.048	0.640	0.188	0.162
Full	3.000	0.004	−0.004	0.002	0.003	0.003	0.005	0.962	0.938	0.956
New	3.000	0.007	−0.005	0.003	0.005	0.005	0.009	0.950	0.948	0.952
Naive	3.000	0.206	0.215	−0.305	0.050	0.049	0.100	0.265	0.036	0.018
Full	4.000	0.003	−0.007	0.006	0.003	0.003	0.005	0.950	0.944	0.952
New	4.000	0.007	−0.008	0.007	0.006	0.005	0.011	0.956	0.958	0.948
Naive	4.000	0.323	0.274	−0.393	0.114	0.078	0.161	0.038	0.002	0.000
Full	5.000	0.004	−0.004	0.000	0.004	0.003	0.005	0.934	0.956	0.948
New	5.000	0.013	−0.009	0.006	0.006	0.007	0.013	0.958	0.952	0.950
Naive	5.000	0.498	0.321	−0.467	0.264	0.106	0.224	0.000	0.000	0.000

The results are similar to those for the smaller sample size. The bias and mean-square error are similar to those in Table 2, part (a), for all three parameters in the naive method but the bias, mean-squared error and coverage for our likelihood method and the full data become very close with increased sample size. The coverage for the naive method becomes worse, since the standard errors become smaller with increased sample size, but the bias is of order 1.

This simple simulation study illustrates that the methodology in Section 2 is of value primarily when explanatory variables are time varying. Furthermore, the effect of ignoring updates of such variables that are available from visits for which an outcome measure is not taken depends on the extent of mismeasurement of the explanatory variables that this induces.

## 4. Informative observation

The previous section was based on an implicit assumption that the pattern of the missing count data was not informative and thus missing at random. To generalize the approach, the observed data can be regarded as coarsened data (Heitjan and Rubin, 1991) in the sense that what is observed is a subset of the complete-data sample space that is defined by all observed and unobserved *Y*_*i*,*j*_-values. A similar view was adopted by Shardell *et al.* (2007) for longitudinal observation of discrete event time data. Non-informative observation can then also be termed coarsening at random.

To allow coarsening not at random, we require some model of the observation process. Let *G*_*i*,*j*_ be a variable that describes the level of coarsening. Further, let *h*{*g*_*i*,*j*_|*y*_*i*,*j*_,*x*_*i*,*j*_,*z*(*d*_*i*,*j*_),***γ***} be the probability function for *G*_*i*,*j*_ conditional on the joint counts and explanatory variables at visit *j* and with unknown parameters ***γ***. Then the likelihood development of Section 2 will be unchanged except that all occurrences of *f*{*r*,*μ*(*η*),***ψ***} will be replaced by a product of the form *f*{·} *h*{·}.

We let *G*_*i*,*j*_ be a binary variable that takes the value 1 if the increment in damaged joint count *Y*_*i*,*j*_ is observed and 0 otherwise; hence 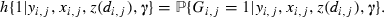


In principle, the observation process could be modelled in other manners, e.g. by a model for the width of the interval in which *Y* is observed to lie. Again, this could be simply incorporated into the development of Section 2. However, when considering informative observation in the following section, we shall restrict attention to a binary observation variable and further simplify, for illustration purposes, by allowing observation to depend only on the increment in the joint count *Y*. It is convenient then to adopt the logistic model 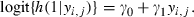
(4)

This is the selection model for a dropout process that was suggested by Diggle and Kenward (1994) where a non-zero value for the regression coefficient *γ*_1_ corresponds to a departure from an assumption of missingness at random. The identifiability of such models is highly dependent on distributional assumptions (Fitzmaurice *et al.*, 1996). Also, the effect of missingness that depends only on outcome is expected to be limited with discrete data, being non-existent in the case of logistic regression (Farewell, 1979). We adopt the model represented by equation (4) here simply to illustrate the potential to extend the methods in Section 2. Adapting the methods to pattern–mixture models (Little, 1993) would also be of interest.

## 5. Application

The starting point for the results that are reported here was the negative binomial model that was presented by Bond *et al.* (2007) in their Table 4. The use of a negative binomial model is motivated by overdispersion in the data, particularly linked to an excess of 0s compared with a Poisson model. The model was developed from the psoriatic arthritis data that were introduced in Section 1. A summary of the characteristics of the patients at their initial clinic visits is given in Table 3. The model was for the increment in the number of damaged joints, determined clinically, between clinic visits. On the basis of a process of variable selection, it modelled the relationship between the log-relative-damage rates and explanatory variables: sex, age, time in clinic, initial erythrocyte sedimentation rate (ESR) current number of tender joints, current number of swollen joints, medication (none, non-steroidal anti-inflammatory drugs (NSAIDs), disease modifying anti-rheumatic drugs (DMARDs) and steroids). These correspond to the explanatory variables **X** as defined in Section 2 and most of these variables are time varying so there is the potential for bias in the estimation of regression coefficients if outcome data are not available at each clinic visit. The model also estimated relative rates that are associated with the current damage count (corresponding to the dynamic covariates **Z**(*D*) which were referred to in Section 2), in particular with a categorization of the damage count (0, [1–4], [5–9] and greater than 9), and an interaction variable being the product of the length of the disease and an indicator of whether the patient has any damaged joints. The offset term *O*_*j*_ was the logarithm of the time interval between observations *j* and *j*+1; hence the coefficients estimate the relative damage *rate* (on a log-scale). Here we consider models with the same explanatory variables and dynamic covariates as chosen by Bond *et al.* (2007) but which are estimated from different subsets of the data.

**Table 4:** Estimated log-relative-risks and dispersion (***β***_*X*_, ***β***_*Z*_, *θ*) for model (a)

*Characteristic*	*Estimate*	*Standard error*	*Level of significance*
Intercept	−8.6669	0.3257	<10^−6^
Sex (male)	0.0174	0.1170	0.8819
Age (per year)	0.0078	0.0048	0.1062
Initial ESR	0.0088	0.0026	0.0007
Initial effused joint count			
1–4	0.1375	0.1280	0.2825
5–9	0.1163	0.1775	0.5126
≥10	0.0912	0.2426	0.7069
Current tender joint count (per joint)	0.0211	0.0094	0.0247
Current effused joint count (per joint)	0.0881	0.0187	<10^−6^
Time in clinic (per year)	−0.0408	0.0106	0.0001
Medication			
DMARDs	0.1888	0.2107	0.3704
NSAIDs	−0.2206	0.2591	0.3944
Steroids	0.4224	0.2296	0.0658
Current deformed joint count			
1–4	1.2164	0.1771	<10^−6^
5–9	1.8182	0.2133	<10^−6^
≥10	1.8901	0.1987	<10^−6^
Initial arthritis duration (per year)			
Current deformed joint count=0	0.0315	0.0144	0.0284
Current deformed joint count > 0	0.0071	0.0089	0.4243
Dispersion (*θ*)	0.1753	0.0104	<10^−6^

**Table 3:** Baseline characteristics of the data

*Characteristic*	*Value*
Number of patients	625
Median number of clinic visits (range)	7 (2, 76)
Female/male	272/353
Median age, years (range)	34 (9, 86)
Median duration of arthritis years (range)	4.5 (0, 47.7)
Median number of tender joints (all joints) (range)	4 (0, 43)
Median number of tender joints (hands and feet) (range)	3 (0, 35)
Median number of swollen joints (all joints) (range)	2 (0, 33)
Median number of swollen joints (hands and feet) (range)	1 (0, 28)
Median ESR (range)	22.5 (0, 105)
Damaged joints (all joints)	
None	62.2% (389)
1–4	20.8% (130)
5–9	5.9% (37)
>9	11.1% (69)
Damaged joints (hands and feet)	
None	68.3% (427)
1–4	17.3% (108)
5–9	5.0% (31)
>9	9.4% (59)
Medication	
None	24.3% (152)
NSAIDs	30.6% (191)
DMARDs	40.5% (253)
Steroids	4.6% (29)

The specific form of the model can be written as 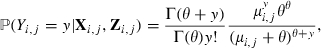
(5)
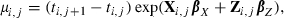
(6) where **X**_*i*,*j*_ and **Z**_*i*,*j*_ are row vectors of the covariates described above for patient *i* at visit *j*, which occurred at time *t*_*i*,*j*_; ***β***_*X*_ and ***β***_*Z*_ are vectors of coefficients to be estimated and *θ* is the dispersion parameter to be estimated.

Our analysis incorporates the interpatient correlation of the observations from one patient through the use of dynamic covariates, i.e. the current damage count and a related interaction. Aalen *et al.* (2004) have discussed the practical use of this approach. Alternative routes could include using random effects (McCulloch and Searle, 2001), or using generalized estimating equations (Liang and Zeger, 1986). These are discussed briefly in Section 6. Use of the current damage count has the practical advantage of being immediately interpretable to a clinical audience. The implicit assumption is that, conditional on the current damage count, the next increment is independent of previous increments. In previous work (Bond *et al.*, 2007), the model that is represented by equations (5) and (6) was fitted and standard errors of the regression coefficients were estimated by using the generalized estimating equation methodology with an exchangeable working correlation. The estimates and standard errors were very similar to those obtained by using regular maximum likelihood estimation. This is consistent with Cox and Snell (1981), page 23, who advised that it is sensible to be most critical about primary aspects of a problem but simplifying assumptions can be made with regard to secondary aspects. Here, primary interest is in the marginal effects of the explanatory variables, and *some* reasonable attempt to capture any correlation structure should be adequate.

We examine a series of models that should produce roughly comparable estimates.

- The first model is based on the clinical damage counts for joints in the hands and feet and uses data from all clinic visits. Here this can be regarded as the ‘correct’ model since it is based on all the relevant information. It is fitted by using standard software.
- This model takes the data that are used in model (a) and deletes *all* the variables on visits where no radiological observations were made. Estimates are obtained by using standard software, ignoring the updated covariates. Hence it is the naive method of estimation.
- This model takes the data that are used in model (a) but deletes *just the outcome variable* for visits where no radiological observations were made. Estimates are obtained by using our method where the covariates that depend on the unobserved outcome variable, **Z**(*D*), are calculated as in equation (3).
- The penultimate model uses the radiological damaged joint counts and a comparison of it with model (c) provides an indication of how model-based conclusions might differ depending on whether clinical or radiographical damaged joint counts are used as the outcome variable.
- The final model generalizes model (d) by incorporating a logistic model for the probability of a radiological count being available in the form of equation (4).

**Fig. 1:**
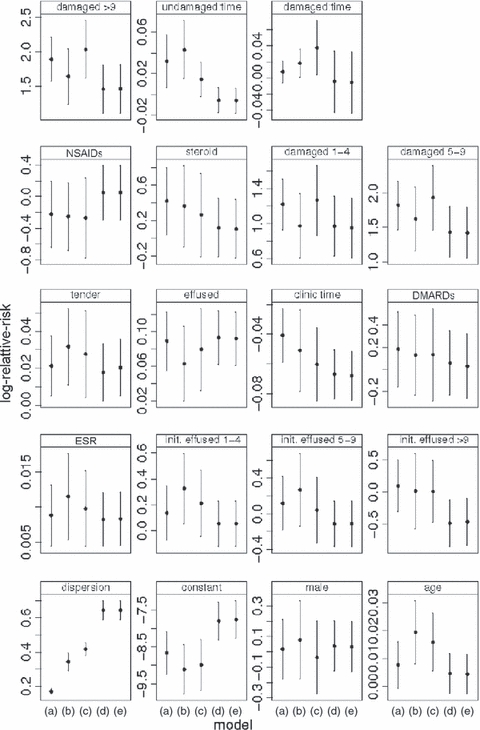
Comparison of estimated parameters and 95% confidence intervals: models (a)–(e) are from left to right

Table 4 shows the results of fitting model (a). The regression coefficients reflect the importance of the various explanatory variables on the development of clinical damage. Fig. 1 then shows 95% confidence intervals and estimates for the 18 regression coefficients, the elements of ***β***, and the dispersion parameter for models (a)–(e). Each box refers to a different regression coefficient and there are five error bars for each of the five models that were fitted. There are no obvious patterns in the regression coefficients, either in terms of the estimates or the standard deviations. The dispersion parameter does appear to become larger as the amount of data that is used becomes less and therefore more uncertainty is introduced. It is also larger for the model based on radiological damaged joint counts, model (d), than for the comparable model based on clinical damage, model (c). Comparison of the lengths of the confidence intervals from models (b) and (c) indicates that there is no consistent pattern across regression coefficients.

The logistic parameters, *γ*_0_ and *γ*_1_, in model (e) were estimated as −1.06 and 0.34 with standard errors 0.04 and 0.03. Thus there is highly significant evidence that observation depends on the increase in damaged joint count. From Fig. 1, however, it is clear that the coefficients that are estimated for models (d) and (e) are very similar. Thus, for these data, little bias appears to have been introduced by the adoption of a coarsening at random assumption in model (d).

From this example, no consistent evidence emerges that the more frequent updating of explanatory variable information that is enabled by the likelihood methodology of Section 2 is dramatically superior to simpler analyses. In addition, our estimates broadly agree with previous studies of these data (Gladman and Farewell, 1999; Gladman *et al.*, 1994).

## 6. Discussion

In this paper we have illustrated how to implement likelihood-based inference for a regression model for longitudinal data when an outcome variable is only partially observed. The method is applicable to maximum likelihood estimation when the outcome variable is the increment in a cumulative, discrete quantity that can be missing at specific times of observation but when the time varying explanatory variables are available at all observation times. Generalization to account for informative observation is relatively straightforward. The choice of probability distribution is arbitrary as long as the sample space is discrete.

As pointed out by a referee, in the general context of regression models for longitudinal data, we cannot expect to improve a likelihood-based complete-case analysis if outcomes are missing but missingness at random holds. Here, the situation is special in that, in spite of the missing data, there is some information on the outcome because the sum over several outcomes is known.

In the example, our choice of the negative binomial can be regarded as a Poisson distribution with a gamma-distributed multiplicative random effect. We could generalize the assumption of conditional independence between increments, given current damage counts, by use of a Poisson model with a gamma-distributed random effect that is common within a patient. To use the multistate model framework to cope with the missing outcomes, we would have to factorize the joint likelihood of *all measurements* on a patient, which is available in a closed form, into a product of conditional likelihoods: 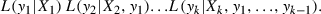


After some algebra, it can be shown that 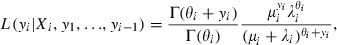 which is very similar to equations (5) and (6), with equation (6) remaining the same, but with *θ*_*i*_ and *λ*_*i*_ obeying the recursive relationships 
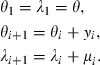


Hence such a random-effects model could be implemented in principle and is a topic for future research.

In contrast, it is not apparent how to combine the multistate model approach to the missing outcomes, which is a full likelihood approach, with a *generalized* estimating equation approach (Liang and Zeger, 1986) that uses a working correlation matrix other than the independent correlation matrix. Even the use of a working independence assumption is computationally complex. Hence we have not pursued the generalized estimating equation approach.

Our method was developed in response to a specific application: the analysis of data from psoriatic arthritis patients. In this setting, the uncertainty in how best to measure joint damage required the analysis of radiological damage information which is not observed at each clinic visit. Our software was written by using the R programming language (Ihaka and Gentleman, 1996) and can be downloaded from. The download also contains a subset of the data consisting of the response and a few of the covariates. Computational efficiency might be improved with alternative programming packages.

Our simulation studies focused on the negative binomial distribution, which is suitable for count data with overdispersion. It shows that the naive method that ignores the missing observations is biased in estimating regression coefficients when the explanatory variables vary over the observations with missing outcomes. This bias does not improve with larger sample size. However, our likelihood method is consistent and has accurate confidence interval coverage error.

The practical implications of the incorporation of updated explanatory variable information may vary from application to application, depending primarily on the strength of relationships with explanatory variables and, most importantly, the amount of variation over time in the explanatory variables. With our psoriatic arthritis data, the effect was shown to be minimal, which is itself of considerable importance in strengthening the findings of Bond *et al.* (2007) that demonstrated that the use of clinical measures of damage provided similar information regarding progression of disease as would be provided by the use of radiological measures. Consideration of other realistic settings with comparable characteristics would be informative.
